# Caloric restriction rescues yeast cells from alpha-synuclein toxicity through autophagic control of proteostasis

**DOI:** 10.18632/aging.101675

**Published:** 2018-12-07

**Authors:** Belém Sampaio-Marques, Hélder Pereira, Ana R. Santos, Alexandra Teixeira, Paula Ludovico

**Affiliations:** ^1^Life and Health Sciences Research Institute (ICVS), School of Medicine, University of Minho, Braga, Portugal; ^2^ICVS/3B’s - PT Government Associate Laboratory, Braga/Guimarães, Portugal; * Equal contribution

**Keywords:** caloric restriction, aging, ubiquitin-proteasome system, autophagy, alpha-synuclein

## Abstract

α-Synuclein (SNCA) is a presynaptic protein that is associated with the pathophysiology of synucleinopathies, including Parkinson’s disease. SNCA is a naturally aggregation-prone protein, which may be degraded by the ubiquitin-proteasome system (UPS) and by lysosomal degradation pathways. Besides being a target of the proteolytic systems, SNCA can also alter the function of these pathways further, contributing to the progression of neurodegeneration. Deterioration of UPS and autophagy activities with aging further aggravates this toxic cycle. Caloric restriction (CR) is still the most effective non-genetic intervention promoting lifespan extension. It is known that CR-mediated lifespan extension is linked to the regulation of proteolytic systems, but the mechanisms underlying CR rescue of SNCA toxicity remain poorly understood. This study shows that CR balances UPS and autophagy activities during aging. CR enhances UPS activity, reversing the decline of the UPS activity promoted by SNCA, and keeps autophagy at homeostatic levels. Maintenance of autophagy at homeostatic levels appears to be relevant for UPS activity and for the mechanism underlying rescue of cells from SNCA-mediated toxicity by CR.

## Introduction

α-Synuclein (SNCA) is a presynaptic protein and the major component of Lewy bodies, the hallmark proteinaceous inclusions implicated in synucleinopathies, including Parkinson’s disease. While it is evident that SNCA is a key player in synucleinopathies pathogenesis, its exact role, although broadly investigated, is not yet fully understood, either in health or in disease [1].

Proteostasis (protein homeostasis) is maintained by a fine balance between folding, refolding and/or degradation of proteins, orchestrated by molecular chaperones and the proteolytic systems: the ubiquitin-proteasome system (UPS) and lysosome-mediated degradation pathways (reviewed in [2]). SNCA, an aggregation-prone protein, is a target for degradation by proteolytic systems including UPS and macroautophagy (hereafter called autophagy) (reviewed in [3-6]). However, the pathways of SNCA degradation remain controversial and the mechanism(s) that govern whether SNCA will be degraded by the UPS or autophagy remain(s) unclear [4]. In yeast cells expressing SNCA, the pharmacological and genetic inhibition of proteasome resulted in contradictory observations [7-11]. Nonetheless, both degradation pathways, UPS and autophagy, become overloaded in pathological conditions associated with synucleinopathies, such as those caused by SNCA overexpression or the presence of point mutations [7,12-14]. Failure of SNCA clearance due to impairment of protein quality control systems, particularly autophagy, has become a recognized central and common pathogenic event of synucleinopathies [4,12,15-17]. This event of proteostasis collapse is aggravated by aging, a major risk factor for neurodegenerative disorders [18]. Thus, interventions that delay aging could have promising therapeutic effects in PD and other synucleinopathies.

Caloric restriction (CR) is still one of the most effective non-genetic interventions known to promote lifespan extension in several model organisms [19]. CR was shown to alleviate SNCA toxicity in different PD models, ranging from the unicellular yeast to *Caenorhabditis elegans*, mice and primates [20-25]. CR has been shown to ameliorate the age-related decline of proteostasis by the activation of the protein degradation machinery or through alterations in the balance between protein synthesis and degradation (reviewed in [26]). With regard to protein degradation, CR is known as an enhancer of both UPS [27-29] and autophagy, the later through the modulation of different regulatory pathways/molecules, such as TORC1 or AMPK (yeast homologue of Snf1). However, how CR impacts on the crosstalk between UPS and autophagy and its contribution for SNCA toxicity, a critical stress scenario in aging conditions, remains mostly unexplored. Herein, using a simple and powerful model organism, the yeast *Saccharomyces cerevisiae* heterologously expressing the human SNCA, we aimed to understand the contribution of proteolytic systems and their crosstalk to the beneficial effects promoted by CR intervention during aging.

Our data shows that CR promotes the upregulation of UPS accompanied by the maintenance of autophagy at homeostatic levels in aged SNCA-expressing cells. The study of the crosstalk between UPS and autophagy indicated that UPS is increased upon autophagy inhibition, but autophagy is not enhanced by UPS inhibition under CR conditions. These results suggest an important role of autophagy on regulation of proteasome homeostasis under CR conditions. Maintenance and regulation of autophagy at homeostatic levels appears to be the central mechanism underlying the cell’s rescue from SNCA-mediated toxicity by CR.

## RESULTS

### Caloric restriction enhances ubiquitin-proteasome activity of aged SNCA-expressing cells

**Figure 1:**
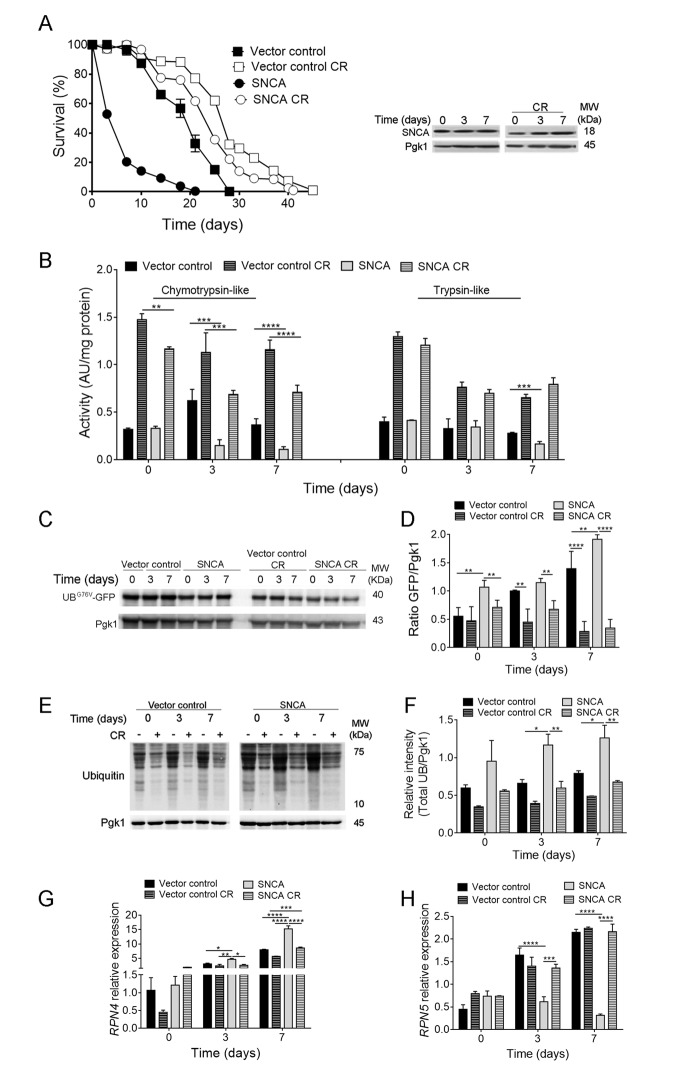
**Caloric restriction abrogates α-synuclein (SNCA)-induced toxicity by upregulating ubiquitin-proteasome system activity.** (**A**) Chronological lifespan (CLS) and SNCA levels of stationary wild type cells harbouring the vector control or expressing the human SNCA grown under regular (2% glucose) or CR (0.5% glucose) conditions. (**B**) Chymotrypsin- and trypsin like activities. The assay was normalized to the total protein amount. (**C**) UPS activity measured by monitoring the ubiquitin/proteasome-dependent proteolysis of the short-lived protein UB^G76V^-GFP. GFP was detected by Western blotting using a GFP-specific antibody. (**D**) Graphical representation of GFP/Pgk1 obtained by densitometric analysis. (**E**) Ubiquitination profile determined by Western blotting using an anti-mono and polyubiquitination antibody. (**F**) Graphical representation of the intensity of total UB/Pgk1 obtained by densitometric analysis. (**G**) *RPN4* and (**H**) *RPN5* mRNA relative expression levels. Three reference genes (*ACT1*-actin, *PDA1*-alpha subunit of pyruvate dehydrogenase and *TDH2*-isoform 2 of glyceraldehyde-3-phosphate dehydrogenase) were used as internal standards and for the normalization of mRNA expression levels. Significance was determined by two-way ANOVA (*p≤0.05, **p≤0.01, ***p≤0.001, ****p≤0.0001) between cells grown under regular or CR conditions expressing vector control or SNCA. Data represents mean ± SEM of at least three biological independent replicas. The error bars represent the standard error of the mean (SEM).

Our previous results showed that caloric restriction (CR) promotes chronological lifespan (CLS) extension of yeast cells heterologously expressing the human α-synuclein (SNCA) (Fig. 1A) associated with the maintenance of autophagy at homeostatic levels [21]. CR promoting-longevity effects are highly dependent on the main proteolytic systems, ubiquitin-proteasome system (UPS) and autophagy, both affected by aging and SNCA expression. Thus, the contribution of these two cellular processes to CR-extended longevity in SNCA-expressing cells was evaluated. UPS activity, namely the chymotrypsin- and trypsin-like activities, was assessed during CLS. The results showed that SNCA affects UPS functioning by leading to a dramatic reduction of chymotrypsin-like activity in aged cells (Fig. 1B). Trypsin-like activity was affected only after 7 days of CLS (Fig. 1B). Decreased UPS activity in SNCA-expressing cells was also confirmed by the increased accumulation of the short-lived protein UB^G76V^-GFP (Fig. 1C and 1D), which reflects a reduction of the ubiquitin/proteasome-dependent proteolysis. These findings were further corroborated by the increase in protein ubiquitination profile (Fig. 1E and 1F) and by the increased *RPN4* relative mRNA expression levels (Fig. 1G). The increase in the *RPN4* mRNA levels in SNCA-expressing cells are accompanied by a decrease in the mRNA levels of *RPN5,* a subunit of the 26S proteasome complex, indicating that the proteasome stress regulon is operating under this proteotoxic conditions (Fig. 1H). Deletion of *RPN4* abolishes the Rpn4-dependent upregulation of *RPN5* transcription (data not shown) as previously described for other stress conditions [30]. These results agree with a previous report showing that SNCA alters proteasome composition and impairs proteasome-mediated protein degradation affecting the survival of stationary phase cells [31].

Importantly, CR intervention promoted an increase of UPS activity (Fig. 1B, 1C and 1D), increase of *RPN5* mRNA levels and reduction of *RPN4* levels, and protein ubiquitination profile (Fig. 1) in SNCA-expressing cells submitted to CR. Comparing the *RPN4* and *RPN5* mRNA levels with UPS activities of caloric restricted SNCA-expressing cells, it is possible to conclude that UPS activity is increased since day 0 while *RPN4* and *RPN5* mRNA levels are only decreased and increased, respectively, at day 3 of CLS (Fig. 1). This is most probably the result of the negative feedback regulation of proteasome homeostasis. Overall the data presented suggests that CR intervention is able to increase UPS functioning during aging of SNCA-expressing cells.

### Autophagy inhibition in SNCA-expressing cells submitted to caloric restriction is associated with increased ubiquitin-proteasome activity

**Figure 2:**
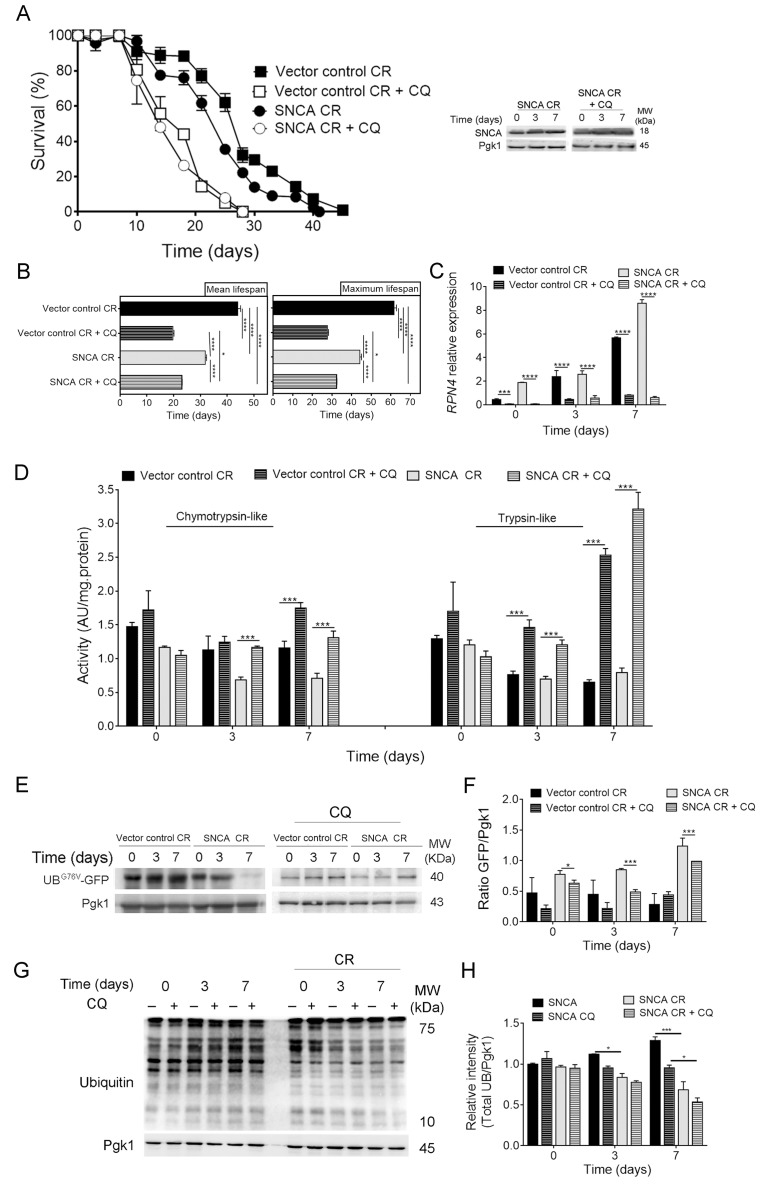
**Autophagy inhibition leads to upregulation of the ubiquitin-proteasome system activity in α-synuclein (SNCA)-expressing cells under caloric restriction.** (**A**) Chronological lifespan (CLS) and SNCA levels of SNCA-expressing stationary wild type cells, under caloric restriction (CR, 0.5% glucose) conditions, in the presence or absence of chloroquine (CQ), an inhibitor of autophagy. (**B**) Mean (50% survival) and maximum (10% survival) lifespans determined from curve fitting of the survival data from CLS. Significance was determined by two-way ANOVA (*p≤0.05, ****p≤0.0001) between cells grown under CR conditions expressing vector control or SNCA in the presence or absence of CQ. (**C**) *RPN4* mRNA relative expression levels as described in the legend of Figure 1. (**D**) Chymotrypsin- and trypsin like activities. The assay was normalized to the total protein amount. (**E**) UPS activity measured by monitoring the ubiquitin/proteasome-dependent proteolysis of the short-lived protein UB^G76V^-GFP. GFP was detected by Western blotting using a GFP-specific antibody. (**F**) Graphical representation of GFP/Pgk1 obtained by densitometric analysis. (**G**) Ubiquitination profile determined by Western blotting using an anti-mono and polyubiquitination antibody. (**H**) Graphical representation of the intensity of total UB/Pgk1 obtained by densitometric analysis. Statistical significance represented in (**C**), (**D**), (**F**) and (**H**) was determined by Student's t-test (*p≤0.05, ***p≤0.001, ****p≤0.0001) comparing caloric restricted vector control or SNCA-expressing cells in the presence or absence of CQ. Data represents mean ± SEM of at least three biological independent replicas. The error bars represent the standard error of the mean (SEM).

The crosstalk between UPS and autophagy is known to be critical for the surveillance and prevention of protein misfolding toxicity [26,32,33]. Our data showed that CR is able to modulate both UPS (Fig.1) and autophagy [21,34] in aged SNCA-expressing cells, suggesting a coordination between both in this scenario. To explore this hypothesis, autophagy was inhibited in CR conditions and the response of SNCA-expressing cells evaluated during aging. Inhibition of autophagy with chloroquine (CQ) in caloric restricted cells expressing SNCA resulted in a shorter CLS, as compared with cells expressing vector control in the same conditions (Fig. 2A and 2B). These results were further supported by the comparison of the mean (50% of survival) and maximum (10% of survival) lifespans of SNCA-expressing cells with vector control expressing cells, under CR in the presence or absence of CQ (Fig. 2B). These data suggest a relevant role of autophagy in CR-mediated lifespan extension of SNCA-expressing cells. Although autophagy inhibition resulted in a decreased CLS of caloric restricted SNCA-expressing cells (Fig 2A), the CLS of these cells is still increased when compared to SNCA-expressing cells under normal growth conditions (2% glucose) (mean lifespan of 23.1 ± 0.2 days versus 7.7 ± 0.6 days) (Fig. 1A). Next, we wondered about the contribution of UPS activity for the CR effects on the CLS of SNCA-expressing cells when autophagy is inhibited. Pharmacological inhibition of autophagy resulted in increased UPS activity along chronological aging as reflected by chymotrypsin- and trypsin-like activities (Fig. 2D) and by the decreased levels of UB^G76V^-GFP (Fig. 2E and 2F) in caloric restricted SNCA-expressing cells. The increase of UPS activity elicited by autophagy inhibition is further confirmed by a decrease of the protein ubiquitination profile (Fig. 2G and 2H) and of *RPN4* mRNA levels in SNCA-expressing cells under CR conditions (Fig. 2C). Altogether, the data revealed that UPS and autophagy crosstalk under CR with increased UPS activity as a response to autophagy inhibition.

### Ubiquitin-proteasome activity inhibition does not impact on autophagy of SNCA-expressing cells under caloric restriction

**Figure 3:**
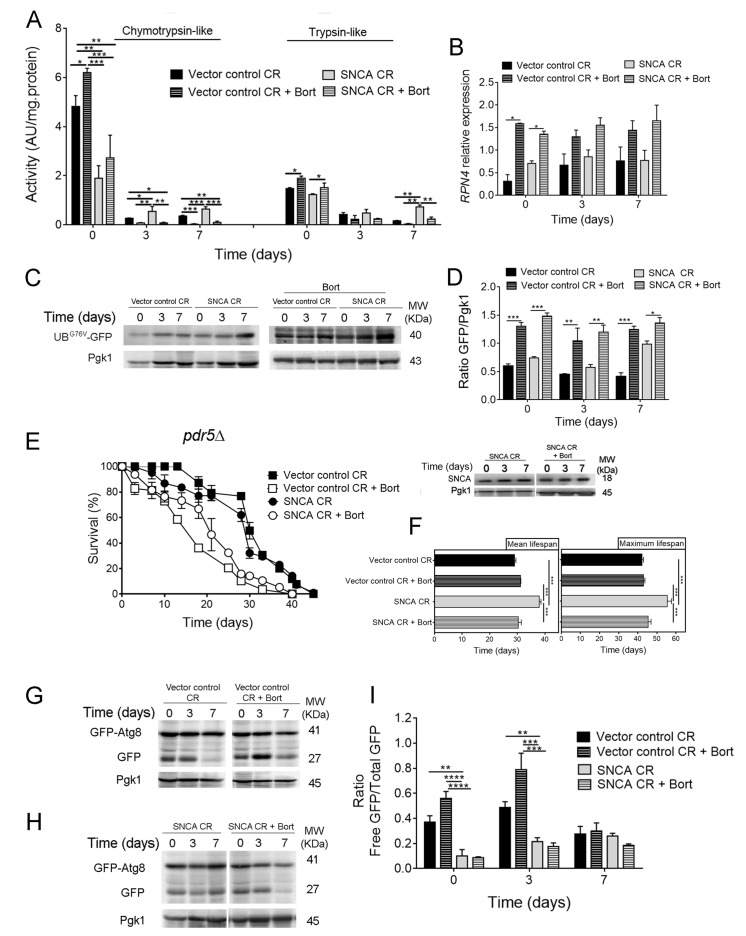
**Pharmacological inhibition of the ubiquitin-proteasome system activity decreases lifespan of α-synuclein (SNCA)-expressing cells grown under caloric restriction but has no major impact on autophagy.** (**A**) Chymotrypsin- and trypsin like activities. The assay was normalized to the total protein amount. Significance was determined by two-way ANOVA (*p≤0.05, **p≤0.01, ***p≤0.001) between cells grown under CR (0.5% glucose) conditions expressing vector control or SNCA in the presence or absence of bortezomib (Bort). (**B**) *RPN4* mRNA relative expression levels as described in the legend of Figure 1. (**C**) UPS activity measured by monitoring the ubiquitin/proteasome-dependent proteolysis of the short-lived protein UB^G76V^-GFP. GFP was detected by Western blotting using a GFP-specific antibody. (**D**) Graphical representation of GFP/Pgk1 obtained by densitometric analysis. Statistical significance represented in (**B**) and (**D**) was determined by Student's t-test (*p≤0.05, **p≤0.01, ***p≤0.001) comparing caloric restricted vector control or SNCA-expressing cells in the presence or absence of Bort. (**E**) Chronological lifespan (CLS) and SNCA levels of *pdr5Δ* cells expressing SNCA grown under CR conditions, in the presence or absence of Bort. (**F**) Mean (50% survival) and maximum (10% survival) lifespans determined from curve fitting of the survival data from CLS. Significance was determined by two-way ANOVA (***p≤0.001) between cells grown under CR conditions expressing vector control or SNCA in the presence or absence of Bort. Autophagy flux assessed by the GFP-Atg8 processing assay (immunoblotting analysis with antibody against GFP) of caloric restricted cells expressing vector control (**G**) or SNCA (**H**) in the absence or presence of Bort. Blots represented in (**G**) are from the same gel, as in (**H**). (**I**) Densitometric analysis of the ratio between the free GFP versus the total GFP. Bands were quantified by Quantity One software. Significance of the data was determined by two-way ANOVA (**p≤0.01, ***p≤0.001, ****p≤0.0001) between cells grown under CR conditions expressing vector control or SNCA in the presence or absence of Bort. Data represents mean ± SEM of at least three biological independent replicas. The error bars represent the standard error of the mean (SEM).

To further explore the crosstalk between autophagy and UPS in SNCA-expressing cells under CR conditions, UPS activity was pharmacological inhibited with two different proteasome inhibitors, bortezomib and MG132. The use of proteasome inhibitors in *S. cerevisiae* cells requires reduced drug efflux that is achieved by deletion of *PDR5*, a multidrug transporter involved in pleiotropic drug resistance [35]. Thus, *pdr5Δ* cells heterologous expressing SNCA were cultured under CR conditions and treated with proteasome inhibitors. The pharmacological inhibition of UPS activity was confirmed by the drastic reduction of chymotrypsin- and trypsin-like activities (Fig. 3A) and by the accumulation of UB^G76V^-GFP, promoted by bortezomib (Fig. 3C and 3D) and MG132 (data not shown), in SNCA-expressing cells under CR. The inhibition of UPS activity is also supported by the increased *RPN4* mRNA levels (Fig. 3B).

Inhibition of UPS activity either with bortezomib (Fig. 3) or MG132 (data not shown) resulted in a shorter CLS, corresponding to decreased mean and maximum lifespans, of SNCA-expressing cells under CR conditions when compared to vector control expressing cells (Fig. 3E and 3F). These results indicate that UPS is also an important component of CR-mediated longevity. Autophagy was also evaluated in caloric restricted SNCA-expressing cells in the presence of proteasome inhibitors. Notably, the results obtained for autophagy, showed that proteasome inhibition has no major impact on autophagy flux in SNCA-expressing cells under CR conditions (Fig. 3G, 3H and 3I).

**Figure 4:**
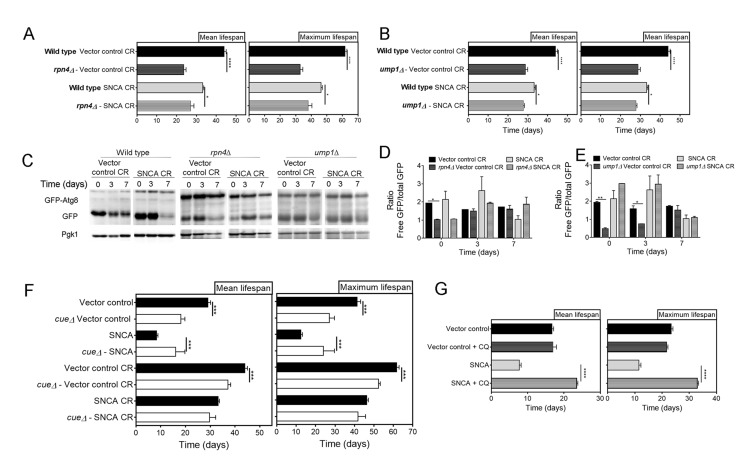
**Genetic inhibition of the ubiquitin-proteasome system activity decreases lifespan of α-synuclein (SNCA)-expressing cells grown under caloric restriction conditions but has no major impact on autophagy.** Mean (50% survival) and maximum (10% survival) lifespans determined from curve fitting of the survival data from CLS of caloric restricted vector control and SNCA expressing wild type cells compared with *RPN4* (**A**) and *UMP1* (**B**) deleted cells. (**C**) Autophagy flux assessed by the GFP-Atg8 processing assay (immunoblotting analysis with antibody against GFP) of wild type, *rpn4*Δ and *ump1*Δ caloric restricted cells expressing vector control or SNCA. Blots from same strain were run in the same gel. Densitometric analysis of the ratio between the free GFP versus the total GFP of *rpn4*Δ (**D**) and *ump1*Δ (**E**) cells. Data from wild type cells is repeated in (**D**) and (**E**) for easier interpretation. Bands were quantified by Quantity One software. (**F**) Mean and maximum lifespans determined from curve fitting of the survival data from CLS of wild type and *cue5*Δ cells expressing the vector control or SNCA in normal (2% glucose) or CR (0.5% glucose) growth conditions. (**G**) Mean and maximum lifespans determined from curve fitting of the survival data from CLS of wild type cells expressing the vector control or SNCA in the absence or presence of chloroquine (CQ). Significance was determined by Student's t-test (*p≤0.05, **p≤0.01, ***p≤0.001, ****p≤0.0001) comparing wild type with each mutant strain in the same conditions. For (**G**) the comparison was done between vector control or SNCA-expressing cells in the presence or absence of CQ. Data represents mean ± SEM of at least three biological independent replicas. The error bars represent the standard error of the mean (SEM).

UPS activity was also genetically inhibited by deletion of *UMP1* or *RPN4*. *UMP1* deletion causes incorrect assembly of the 20S proteasome and reduced ubiquitin-mediated proteolysis [36], while *RPN4* is a transcriptional regulator of the 26S proteasome [37]. Both mutants were confirmed to have impaired UPS activity (data not shown). The comparison of the mean and maximum lifespans of *RPN4* and *UMP1* cells expressing vector control or SNCA with the correspondent wild type cells revealed that genetic abrogation of UPS activity reduces CR-mediated extension of CLS (Fig. 4A and 4B). These results are in agreement with the observations made with pharmacological inhibition of UPS (Figure 3), pointing, once again, UPS as a relevant factor of CR-mediated longevity. Regarding autophagy, the results showed that genetic inhibition of UPS activity, by abrogation of *RPN4* or *UMP1*, does not have a major impact on autophagy flux of caloric restricted cells expressing SNCA (Fig. 4C, 4D and 4E), as the pharmacological inhibition of UPS activity (Fig. 3).

Autophagy has an important role on the regulation of proteasome homeostasis by proteophagy, a mechanism dependent on the ubiquitin receptor Cue5, which recognizes inactivated ubiquitylated proteasomes [38]. To elucidate the role of proteophagy on the crosstalk between UPS and autophagy in caloric restricted SNCA-expressing cells, we have used *CUE5* deleted cells. The CLS analysis of wild type and *cue5*Δ cells expressing SNCA revealed a significant increase of mean and maximum lifespans of *cue5*Δ cells (Fig. 4F). However, under CR conditions this effect was abrogate and no differences were observed between the mean and maximum lifespans of wild type and *cue5*Δ cells expressing SNCA (Fig. 4F). As previously described [38], *cue5*Δ cells display decreased autophagy flux (data not shown). The results obtained with *cue5*Δ and wild type cells treated with CQ and/or under CR conditions (Fig. 2, 4F and 4G), suggest that the maintenance of autophagy at homeostatic levels, both in normal growth or CR conditions, is beneficial for UPS activity and reduces SNCA toxicity.

## DISCUSSION

Maintenance of proteostasis and “cleansing” of misfolded proteins is mainly promoted by two proteolytic systems: the ubiquitin-proteasome system (UPS) and lysosomal degradation pathways, particularly autophagy. During aging, both systems are target of extensive alterations culminating in loss of proteostasis that is further potentiated by the accumulation of proteins prone to aggregation such as SNCA [18].

Caloric restriction (CR) is a powerful non-genetic manipulation known to extend lifespan in a number of aging models and is also known to delay or rescue toxicity in synucleinopathies models [19-25]. In spite of the recognized role of the UPS and autophagy proteolytic systems in the beneficial effects of CR, the crosstalk between them remains poorly understood. The data presented herein investigated this crosstalk by individually inhibiting both proteolytic systems in an aging cellular model for synucleinopathies. The results obtained indicate that CR-promoted beneficial effects on aged SNCA-expressing cells are dependent on both UPS and autophagy, in agreement with previous observations in other aging models [27-29,39,40]. The dependency of CR-mediated longevity on proteolytic systems is confirmed by data showing a shorter chronological lifespan when each of these proteolytic systems, UPS and autophagy, is inhibited. Importantly, crosstalk and coordinated regulation of UPS and autophagy are observed when autophagy is pharmacologically inhibited resulting in an increase of UPS functioning. Autophagy inhibition and CR-mediated enhancement of UPS in SNCA-expressing cells are associated with an increase of both chymotrypsin- and trypsin-like activities, while in the literature, a central role in the protein degradation by proteasome is assigned mainly to chymotrypsin-like activity [41,42].

The coordinated regulation of UPS and autophagy seems to be required for protein homeostasis and consequently longevity extension [33,43,44]. The results presented show that pharmacological or genetic inhibition of proteosome in SNCA-expressing cells under CR was not compensated with enhancement of autophagy. This observation seems to contradict the literature showing that autophagy is stimulated to compensate for UPS inhibition [33,43,44]. In stationary phase yeast cells, proteasomes, mainly localized in the nucleus, are translocated to cytosol forming protective proteasome storage granules (PSGs) [45-49]. In yeast PSGs protect proteasomes from autophagic degradation upon carbon starvation [50]. During chronological aging, yeast cells expressing SNCA experience carbon starvation and also an impairment of UPS activity (Fig. 1). Thus, although dysfunctional proteasomes could be degraded by proteophagy [38], degradation is affected because chronological aged cells lack carbon source and thus form PSGs. Our results herein presented show that deletion of the proteophagy selective receptor *CUE5* protects SNCA-expressing cells increasing CLS (Fig. 4F). In addition, CR does not have any effect on the CLS of *cue5*Δ cells expressing SNCA (Fig. 4F). Adding to these findings our previous observations on the impairment of UPS and the drastic increase of autophagy in aged SNCA-expressing cells [34], it is tempting to speculate that the protective effects of CR on SNCA-expressing cells are partially dependent on the inhibition of proteophagy and early protective formation of PSGs. This hypothesis needs further investigations.

Overall, our data suggest that in proteotoxic stress conditions, the CR-mediated beneficial effects are associated with increased UPS activity accompanied by the maintenance of autophagy at homeostatic levels. Furthermore, although under CR conditions inhibition of each proteolytic activity leads to reduced longevity, the maintenance of autophagy at homeostatic levels appears to be the central pathway underlying rescue from SNCA-mediated toxicity.

## MATERIALS AND METHODS

### Strains and media

The yeast strains and plasmids used in this study are listed in Table 1. Cell stocks were maintained in YEPD agar medium containing 0.5% yeast extract, 1% peptone, 2% agar, and 2% glucose. All experiments were performed in synthetic complete (SC) medium containing glucose as a carbon source and 0.67% yeast nitrogen base without amino acids (Difco Laboratories) supplemented with excess amino acids and bases for which the strains were auxotrophic (50 μg/mL histidine, 50 μg/mL lysine, 100 μg/mL methionine, 300 μg/mL leucine and 100 μg/mL uracil).

**Table 1:** Strains and plasmids used in this study.

**Yeast Strain**	**Genotype**

BY4741	*MATa his3Δ1 lys2Δ0 met15Δ0 ura3Δ0*		
BY4741 *pdr5Δ*	*MATa his3D1 leu2D0 met15D0 ura3D0 pdr5::kanMX4*		
BY4741 *ump1Δ*	*MATa his3D1 leu2D0 met15D0 ura3D0 ump1::kanMX4*		
BY4741 *rpn4Δ*	*MATa his3D1 leu2D0 met15D0 ura3D0 rpn4::kanMX4*		
BY4741 *cue5Δ*	*MATa his3D1 leu2D0 met15D0 ura3D0 cue5::kanMX4*		
**Plasmids**		**Type of Plasmid**	**Source**
pYX222-SNCA		2µ	[34]
pRS416-GFPAtg8		2µ	[21]
pYES2-Ub-G76V-GFP		2µ	Addgene

### Chronological lifespan assays

To perform the chronological lifespan (CLS) assays cells were grown on synthetic liquid media with 2% and 0.5% glucose for regular media and caloric restriction conditions, respectively. Overnight cultures were grown in SC medium containing the two different concentrations of glucose and then inoculated into ﬂasks containing medium with the same concentration of glucose at a volume ratio of 1:3. These cultures were then incubated at 26 °C with shaking at 150 rpm. Cultures reached stationary phase 2-3 days later, and this was considered day 0 of CLS. Survival was assessed by counting colony-forming units (CFUs) after 2 days of incubation of culture aliquots at 26 °C on YEPD agar plates beginning at day 0 of CLS (when viability was considered to be 100%) and then again, every 2-3 days until less than 0.01% of the cells in the culture were viable.

Mean (50% survival) and maximum (10% survival) lifespans were determined from curve fitting of the survival data (from pair-matched, pooled experiments) with the statistical software Prism (GraphPad Software).

To block autophagy, 50 µg/µl chloroquine (CQ), or to inhibit UPS activity, 30 µM bortezomib or 50 µM MG132, were added to the culture medium at day 0 of CLS.

### Total protein extraction and western blotting

For detection of protein levels by western blot, total cellular extracts were collected at specific time points and extracted as previously described [51]. Briefly, cells were pre-treated with 2 M lithium acetate for 5 min at room temperature. After lithium acetate removal, 0.4 M NaOH were added for 5 min on ice. Next, the cells were resuspended in SDS-PAGE sample buffer and boiled for 5 min. Total protein quantification was performed with the RC DC protein assay (Bio-Rad) and processed according to the manufacturer’s instructions. Of total protein, 20 μg were resolved on a 12% SDS gel and transferred to a nitrocellulose membrane during 7 min at 25V in Trans-Blot Turbo transfer system. Membranes were blocked with tris buffered saline (TBS) with 0.1% Tween 20 (TBST) containing 5% skim milk, followed by incubation with primary antibodies against: anti-SNCA (1:1000, Sigma); anti-GFP (1:5000; Abcam), anti-mono and polyubiquitination conjugated (1:1000; Enzo) and anti-PGK (1:5000; Invitrogen) in TBST containing 1% BSA and primary antibody. After washing with TBS, the membranes were incubated with the respective secondary antibody, HRP-conjugated anti-rabbit IgG, anti-mouse IgG or anti-goat IgG at a dilution of 1:5000 in 1% skim milk. Protein levels were detected after incubation with SuperSignal West Femto Maximum Sensitivity Substrate (Thermo Scientific) or Clarity Western ECL Substrate (Bio-Rad). Digital images of the western blotting were obtained in a ChemiDoc XRS System (Bio-Rad) with Quantity One (Bio-Rad) software.

### mRNA expression analysis by qRT-PCR

The quantitative mRNA expression analysis was performed according to the MIQE guidelines (Minimum information for publication of quantitative real-time PCR Experiments [52]). Briefly, quantitative real-time PCR (qPCR) was used to measure the mRNA transcripts of the *RPN4* gene (FW: AAGGAAACCAGCAAAATCATC; RV: TTTCTAATGTGCCGTTTTCAT) and *RPN5* gene (FW: TGGCATTTGGTGGAGAAGCT; RV: ATGTTTCCGTCTGGCTCTCC as described in [38]). Three reference genes (*ACT1*-actin (FW: GATCATTGCTCCTCCAGAA; RV: ACTTGTGGTGAACGATAGAT), *PDA1*-alpha subunit of pyruvate dehydrogenase (FW: TGACGAACAAGTTGAATTAGC; RV: TCTTAGGGTTGGAGTTTCTG) and *TDH2*-isoform 2 of glyceraldehyde-3-phosphate dehydrogenase (FW: CCGCTGAAGGTAAGTTGA; RV: CGAAGATGGAAGAGTTAGAGT)) were selected due to their stable expression and were tested in same experimental conditions allowing expression normalization.

Yeast samples for qPCR quantification were centrifuged (4,500 rpm, 4°C, 5 min) and the pellets were immediately stored at -80°C until RNA extraction. The RNA extraction was carried out as previously described [52]. The RNA quality was assessed by agarose gel electrophoresis. Total RNA (300 ng) was reverse-transcribed into cDNA in a 20 μL reaction mixture using the iScript cDNA synthesis kit. Then, 22.5 ng of cDNA of each sample was tested in duplicate in a 96-well plate (Bio-Rad), in a 20 μL reaction mixture using the SsoFast Evagreen Supermix kit (Bio-Rad) and processed according to the manufacturer’s instructions in a CFX96™ Real Time System (Bio-Rad). A blank (no template control) was also incorporated in each assay. The thermocycling program consisted of one hold at 95°C for 1 min, followed by 39 cycles of 15 min at 95°C, 20 s at 57°C and 20 s at 72°C. After completion of these cycles, melting-curve data were collected to verify PCR specificity, contamination and the absence of primer dimers. The PCR efficiency of each primer pair was evaluated by the dilution series method using a mix of sample cDNAs as the template and was determined from calibration curves using the formula 10^(-1/slope)^. Relative expression levels were determined with efficiency correction, which considers differences in the efficiencies between target and reference genes, using the gene expression module of the CFX manager Software (Bio-Rad).

### Proteasome catalytic activity

“Proteasome-Glo TM Cell-Based Assays” (Promega) was used to measure the proteolytic activity of the UPS. This luminescent assay measures the chymotrypsin- and trypsin-like activities associated with the proteasome. Proteasome-Glo Cell-Based Reagents were both prepared and equilibrated at 22°C for 30 min before use. The luminescence was measured in a luminometer “Fluoroskan Ascent FL” by ThermoScientific. About 1x10^6^ cells were plated in a 96-well opaque plate at a volume of 100 µl together with “Proteasom-Glo TM Cell-Based Assays” (100 µl). The assay plates were equilibrated at 22 °C, and after incubation during 5 to 30 min, a luminescent signal was obtained. Protein concentration was used to normalize proteasome activity.

### Monitoring autophagy by GFP-Atg8 processing assay

To perform this assay, cells were transformed with the plasmid pRS416-GFPAtg8 with fusion gene under the control of the *ATG8* endogenous promoter [53,54]. GFP N-terminally tagged to Atg8 is delivered to the vacuole and as GFP is more resistant to degradation than Atg8, bulk autophagy results in the accumulation of free GFP in the vacuole. The ratio between free GFP and total GFP is used as a readout for the autophagic flux. GFP-Atg8 and free GFP are detectable by Western blotting using a GFP-specific antibody (see section Total protein extraction and western blotting). The ratio between free GFP and total GFP is used as a readout for the autophagic flux. Immunoblot bands were quantified by densitometric analysis using the Quantity One software.

### Statistical treatment of data

The results shown are mean values and standard error of the mean of at least three independent assays. Statistical analyses were determined using two-way ANOVA or Student's t-test as indicated in the figure legends. A p-value of less than 0.05 was considered as a signiﬁcant difference.
